# Bacteriology of the Middle Meatus Aspirate in Patients with Cystic Fibrosis

**DOI:** 10.1016/S1808-8694(15)30100-2

**Published:** 2015-10-19

**Authors:** Guilherme Luis da Silva Franche, Fernando Abreu e Silva, Catia de Souza Saleh

**Affiliations:** a Master’s degree in medicine, coordinator of the Rhinology Outpatient Unit, Santa Casa, Porto Alegre.; b Doctoral degree, assistant professor, department of pediatrics and child health care, Rio Grande do Sul Federal University. Head of the pediatric pneumology unit, Clinical Hospital, Porto Alegre.; c ^3^ Medical student, Fundação Faculdade Federal de Ciências Médicas, Porto Alegre.

**Keywords:** bacteriology, cystic fibrosis, middle meatus

## Abstract

The combination of factors, such as abnormal viscosity of the paranasal sinus secretions, decreased sinus drainage, and impaired mucociliary clearance may account for the establishment of a suitable and opportune environment for the colonization of bacteria in the paranasal sinuses of patients with cystic fibrosis.

**Aim:**

The goal of the present study was to assess the bacteriology of the middle meatus aspirate in patients diagnosed whit cystic fibrosis.

**Material and Methods:**

Through a cross-sectional prospective study, a sample consisting of 23 patients evaluated for 2 years, was assessed. Firstly, we established the relationship between the middle meatus culture and the maxillary sinus x-ray. In second, we studied the relationship between the middle meatus aspirate bacteriology and the sputum bacteriology.

**Results:**

In total, 42 aspirates of the middle meatus were carried out. In 17 (73.91%) of the 23 patients, the aspirates were negative; and in 6 (26.08%) they were positive. Out of the 42 aspirates, 31 (78.8%) were negative, and 11 (26.2%) were positive. The presence of Pseudomonas aeruginosa was observed in 18.18% of the positive cultures, and Staphylococcus aureus was observed in 27.28%.

**Conclusion:**

The great majority of the middle meatus aspirates of the patients with cystic fibrosis were negative.

## INTRODUCTION

Cystic fibrosis or mucoviscidosis, is the most frequent genetic autossomal recessive disease in Caucasians.^1^, ^2^ It is a multisystemic, potentially fatal disease that involves primarily epithelial organs.^2^

The incidence varies in different populations. It is about one for each 2,000 live births in Caucasians; it is less frequent in North-American Afro-Americans (1:17,000) and in Asians (1:90,000). According to genetic studies, the incidence of this disease in the city of Porto Alegre is one for each 2,500 live births in Caucasians.^3^, ^4^, ^5^

Clinical findings vary significantly, and most of the organs and body systems may be involved. The triad chronic obstructive pulmonary disease, exocrine pancreatic failure and increased amounts of electrolytes in sweat may be found in most of these patients.^6^

Symptomatic nasal obstruction due to polyp and sinusitis are also found more frequently, given the increased survival of these patients.^7^

Presentation of sinus disease in cystic fibrosis patients is usually subclinical; most of these patients do not have symptoms, although radiograms may show opacification of paranasal sinuses. The most commonly reported complaints are nose blockage and a purulent nasal discharge.^8^

The main aim of this study was to investigate the bacterial cultures of middle meatus aspirates in cystic fibrosis patients. Specific aims were to relate these results with maxillary sinus radiograms, the clinical evaluation and sputum cultures.

## MATERIAL AND METHODS

Following approval from the Research Ethics Committee (protocol number 112/96) a cross-sectional study was made of outpatient cases.

The sample was composed of 23 patients selected from 97 patients that visited the pediatric pneumology outpatient unit during two years and that fulfilled the following inclusion criteria:

a diagnosis of cystic fibrosis based on clinical findings confirmed by two measurements of sodium and chloride over 60mEq/l in sweat; consent to participate after information given to the patient or caretakers; no antibiotic therapy at least one week before the inclusion date; and age over 5 years to simplify the testing procedures.

The middle meatus aspirate for bacterial culture was done with the patient in a sitting position, as follows:

- cotton containing a vasoconstrictor (oxymethazolin) and an anesthetic (neotutocaine 2%) was placed along the lower nasal turbinate for five minutes.
- a Junh-Tym-Tap system and a rigid Storz 2.7 mm diameter 30º angle endoscope connected by cable to a 150-watt halogen light source were used to aspirate secretion. If no secretion was found, the tip of the aspiration system was sent for a bacterial culture.

The aspirated material was sent to the microbiology laboratory of the Porto Alegre Clinical Hospital (HCPA) within 15 minutes and processed routinely for cystic fibrosis patients.

The culture was positive if bacteria reproduced in the following culture media:

- Macconkey (Oxoid Ltd., Hampshire, England)
- Brucella (Becton Dickinson and Company, Cocheysville, USA)
- Azida (Oxoid Ltd, Hampshire, England)
- Agar Chocolate (Becton Dickinson and Company, Cocheysville, USA)
- P Cetrimide Agar (Merck, Darmstadt, Germany)
- PC Burkholderia Cepacia (Mast Diagnostics, Merseyside, UK)

On the same day a radiograph was taken of the maxillary sinuses on the Water’s projection. Radiograms were grouped according to the presence of absence of alterations. Altered radiographies where those with the following findings: air-liquid level, thickening of the mucosa over 6mm and opacification or complete veiling of the maxillary sinus.

Sputum samples were collected by deliberate vigorous coughing, if necessary followed by clapping of the chest; the samples were placed in a sterile flask and taken to the microbiology laboratory of the HCPA within 15 minutes of collection by the author of the current study.

Radiographs were taken in the radiology unit of the HCPA and independently interpreted by radiologists and the author of the current study, none of whom knew the names or clinical histories of patients when reading the radiograph. Agreement was obtained in 100% of cases.

The following symptoms were investigated:

Nasal blockage: patients and their caretakers were asked to report difficulty in nose breathing; if so, whether nose blockage was daily or occasional, in one or both nostrils. Obstruction was diagnosed if the symptom occurred daily.

- Nasal discharge: patients and their caretakers were asked to report frequent nasal discharge. If so, they were asked to describe the color and aspect of the discharge. Nasal discharge was diagnosed if the symptom occurred daily.
- Headache: patients and their caretakers were asked to report headaches. If so, they were asked to describe the site and whether the symptom occurred daily. Headache was diagnosed if the symptom occurred daily.

Anterior rhinoscopy or an examination of the nasal fossae was done using a halogen light photophore and a nasal speculum prior to middle meatus aspiration for bacterial culture. The lower nasal turbinate was considered as enlarged when it took up over 50% of the distance between the septum and the lateral nasal wall. The mucosa of the turbinate was classified as normal, pallid or hyperemic. Secretion was reported, if present, regardless of its aspect. Secretion was classified as serous, mucous, purulent or bloody. Unilateral or bilateral polyps were sought.^9^

The working hypothesis for all of the statistical tests assumed a null relation. The chi-square test or Fisher’s exact test were used to test for the significance of categorical variables. Statistical significance was considered for p<0.05.

Patients and caretakers were informed about the study and gave verbal approval for inclusion. This project is included in category II of the Brazilian Research Regulations for studies in humans (minimal risk). The Research Ethics Committee of the HCPA approved the study.^10^

## RESULTS

The final sample was composed of 23 white patients aged between 5 and 23 years (mean age - 10 years, standard deviation - 4.89 years). There were 16 males (69.6%) and 7 females (30.4%).

Only eight patients (34.8%) reported nasal blockage; 10 patients (43.5%) reported a history of nasal discharge. The aspect of nasal discharges was not stratified, given the difficulty of adequately reporting this finding. The presence of absence of a nasal discharge was noted. No patients reported headaches. There was no relation between the presence of nasal blockage and positive cultures of middle meatus aspirates (p=0.80401). A relation between a history of nasal discharge and positive cultures of middle meatus aspirates, however, was statistically significant (p=0.0000074).

Eight patients (34.8%) presented increased lower turbinates. The middle turbinate mucosa was normal in 22 of 46 nasal fossae. The mucosa was pallid in 16 cases and hyperemic in eight cases. The relation between hyperemia of the middle turbinate and positive cultures of middle meatus aspirates was statistically significant (p=0.0085).

Nasal mucosa secretion was seen in 15 patients (65.2%), classified as serous (53.33%), mucous (26.67%) and purulent (20%). The relation between nasal secretion and positive cultures of middle meatus aspirates was statistically significant (p=0.00272).

Only two patients (8.7%) had nasal cavity polyps, which were bilateral in both cases.

At least one middle meatus was aspirated in all patients to collect secretion. There were 42 aspirates. Cultures were negative in 17 patients (73.91%); cultures were positive in 6 patients (26.08%). Of 42 aspirates, 31 (73.81%) were negative and 11 (26.19%) were positive. In only one patient (4.35%) the culture of middle meatus aspirates was positive for Pseudomonas aeruginosa. The presence of Pseudomonas aeruginosa in positive cultures was 18,18% (table 1).

**Table 1:** Distribution of bacteria in positive cultures of middle meatus aspirates.

BACTERIAS	No	%
Staphylococos aureus	03	27, 28
Pseudomonas aeruginosa	02	18, 18
Haemophylus influenzae	02	18, 18
Streptococcus pneumoniae	02	18, 18
Acinetobacter lwoffi	02	18, 18

Total	11	100, 00

Most of the patients (21 cases - 91.3%) presented altered maxillary sinus radiographies. These were classified as complete opacification of the sinus (78.3%), normal radiography (8.7%), presence of an air-liquid level (8.7%) and presence of mucosal thickening (4.3%). There was no relation between maxillary sinus radiography findings and cultures of the middle meatus (p=0.5583) (Table 2).

**Table 2:** Relation between maxillary sinus radiography and cultures of middle meatus aspirates (p=0.5583).

Maxillary sinus radiography	Culture of the middle meatus	
	Positive	Negative
Altered	11	27
Normal	0	24

Bacterial growth occurred in all of the sputum samples. In seven samples (36.43%) there was growth of two types of bacteria (Table 3). There was no relation between sputum and middle meatus aspirate cultures (p=0.5799) (Table 4).

**Table 3:** Sputum bacterial counts.

Bacterias	No	%
Pseudomonas aeruginosa	12	40, 00
Pseudomonas aeruginosa mucoid	06	20, 00
Staphylococos aureus	07	23, 33
Haemophylus influenzae	02	6, 68
Serratia marcences	01	3, 33
Acinetobacter lwoffi	01	3, 33
Streptococcus pneumoniae	01	3, 33

Total	30	100, 00

**Table 4:** Relation between culture of sputum and culture of the middle meatus (p=0.5799).

Sputum culture	Culture of the middle meatus	
	Positive	Negative
Positive	05	17
Negative	02	03

## DISCUSSION

Although upper airways are frequently involved in cystic fibrosis, there are doubts about whether this condition affects the lower airways.^11^, ^12^, ^13^

Shapiro et al. tested if paranasal sinus mucosal abnormalities in cystic fibrosis patients are merely an adequate surface for bacterial colonization or if bacteria had any significant role in the pathogenesis of tissue damage.^14^

Rhinosinusitis appears to be related to general abnormalities of mucosal secretion. A combination of factors such as paranasal sinus secretion viscosity, reduced sinus drainage, and mucocilliary transport system involvement may be responsible for generating a favorable environment for bacterial colonization in the paranasal sinuses in cystic fibrosis patients.^1^

A high concentration of bacteria in maxillary sinus aspirates from patients with normal radiograms and no specific symptoms suggests that bacterial colonization of the paranasal sinuses is common in these abnormal hosts.^14^

Recent evidence has suggested that there is a higher correlation between endoscopically collected cultures of the middle meatus and of the maxillary sinus. A culture of the maxillary sinus aspirate is the gold standard for the diagnosis of rhinosinusitis.

Orobello et al. compared cultures from the middle meatus with those of the maxillary and ethmoidal sinuses in 39 children and found a correlation of 83% and 80%.^15^

Gold e Tami assessed a group of 18 patients in which they compared middle meatus aspirate cultures obtained by a Juhn-Tym Tap® aspiration system with maxillary sinus aspirate cultures, and found an 85.7% correlation.^16^

Poole found a relation between endoscopically obtained nasal purulent secretion cultures and the typical bacteria of maxillary sinus aspirates.^17^ Bolger’s findings are similar to those of Poole.^18^

Waldya et al. induced acute rhinosinusitis in 24 rabbits and demonstrated a 100% correlation between middle meatus and maxillary sinus cultures.^19^

Although paranasal sinus radiological abnormalities are present in nearly all of the cystic fibrosis patients, few of them present with relevant symptoms.^20^ Paranasal sinus opacification on plain radiograms is probably secondary to mucosal membrane thickening and mucosal gland dysfunction that leads to an increased production of mucus.^13^

Radiography is a poor method for the differential diagnosis of sinus disease, but is sensitive in detecting decreased paranasal cavity transparency; this finding does not always mean a progressive pathological process.^21^, ^22^

In the current series no relation was found between maxillary sinus radiographic findings and the presence of bacteria in the middle meatus aspirate, regardless of the radiological finding.

Since nearly all of the patients had paranasal sinus opacification, it is difficult to assess and establish the impact of sinusitis on cystic fibrosis. Sinus disease together with polyps, however, may significantly decrease the quality of life and the morbidity in these patients.^23^

Pierre Brihaye et al. investigated 84 patients and found nasal blockage (60% of patients), rhinorrhea (53%) and headaches (32%) as the main symptoms. The severity was proportional to nose blockage.^24^

Kerrebijn et al. studied 39 patients that had a mean age of 26 years; 39% of them had a history of frequent or chronic nasal blockage and 30.77% had a history of frequent or chronic nasal discharge within the past six months.^25^ (Table 5).

**Table 5:** Comparison of the clinical history as described in the literature.

	Kerrebijn (Holland)	Brihaye (Belgium)	Franche (Brazil)
Nasal blockage	39%	60%	34, 8%
Nasal discharge	30, 8%	53%	43, 5%
Headache	-	32%	0
Polyps	44%	45%	8, 7%
Mean age	26(17-40)	12 (3 months-34)	10 (5-23)

Here there is a relation between histories of nasal discharge and positive middle meatus cultures.

There was no relation between increased size of the lower turbinate and a middle meatus aspirate positive culture. Both the presence of secretion in nasal fossae and hyperemia of the middle turbinate, however, were significant for middle meatus aspirate positive cultures.

Our findings about middle meatus aspirate cultures are similar to those of Drake-Lee et al., who found positive cultures (33.33%) in 15 needle aspirates of 10 patients. In the positive cultures, 20% were Pseudomonas aeruginosa.^26^ (Table 6 and 7).

**Table 6:** Comparison of bacterial cultures as described in the literature.

	Shapiro (USA-Pittsburg)	Drake-Lee (England)	Franche (Brazil)
Pseudomonas aeruginosa	38, 23%	20%	18, 18%
Staphylococcus aureus	2, 94%	0	27, 28%

**Table 7:** Comparison of maxillary sinus cultures as described in the literature.

	Shapiro (USA-Pittsburg)	Drake-Lee (England)	Franche (Brazil)
Patients	20	10	23
Needle aspiration	34	15	42 (middle meatus)
Mean age (years)	15 (6 a 24)	7, 5 (2 a 15)	10 (5 a 23)
Bacterial growth	76%	33, 33%	26, 19%
Culture	19 (95%)	4 (40%)	6 (26, 08%)

Wiatrak investigated cystic fibrosis patients that underwent surgery and did not find Pseudomonas aeruginosa in cultures. Results were divergent in other studies, however.^27^

In a classical study of 20 patients, Shapiro et al. conducted 34 maxillary sinus needle aspirates and found bacterial growth in 76% of cases. At least one aspirated sinus was positive in 19 of the 20 patients (95%). Although most cultures are positive, only three patients had symptoms that could clearly be associated with sinus disease.^14^ (Table 6 and 7).

Duplechain et al. evaluated 32 children that had undergone nasosinusal endoscopic surgery and found positive cultures for Pseudomonas aeruginosa in 89% of patients in the group of cystic fibrosis patients; the non-cystic group did not have this bacteria.^11^

Umetsu et al. reported Pseudomonas aeruginosa in all of the cultures of 4 patients (mean age - 26 years) that had undergone nasal surgery. All of them had symptoms such as coughing, chronic headaches, nasal blockage and post-nasal discharge. None of them had nasal polyps. The authors concluded that surgical treatment of paranasal sinuses in cystic fibrosis patients might reduce the need for hospitalization, and suggested that nasosinusal disease could exacerbate pulmonary symptoms.^28^

## CONCLUSION

Based on our data we reached the following conclusions:

- Most of the middle meatus aspirate cultures in cystic fibrosis patients were negative.
- There was no relation between maxillary sinus radiographic findings and middle meatus aspirate positive cultures.
- A history of nasal discharge was related with middle meatus aspirate positive cultures.
- Nasal cavity secretion was related with middle meatus aspirate positive cultures.
- Hyperemia of the middle turbinate mucosa was related with middle meatus aspirate positive cultures.
- There was no relation between sputum and middle meatus aspirate culture results.
